# Health Problems and Quality of Life in Patients with Chronic Lower Limb Ischemia—A Polish Population One-Centre-Based Study

**DOI:** 10.3390/healthcare13060643

**Published:** 2025-03-15

**Authors:** Grażyna Bączyk, Katarzyna Anna Kozłowska, Katarzyna Kubiak, Dorota Formanowicz

**Affiliations:** 1 Department of Nursing Practices, Poznan University of Medical Sciences, 61-701 Poznan, Poland; kkozlowska@ump.edu.pl; 2 Vascular Surgery Department, Medical University Clinical Hospital, 61-848 Poznan, Poland; 3 Department of Medical Chemistry and Laboratory Medicine, Poznan University of Medical Sciences, 61-701 Poznan, Poland; doforman@ump.edu.pl

**Keywords:** quality of life, health problems, patients with lower limb ischemia

## Abstract

Background/Objectives: Unhealthy lifestyle and genetic predisposition contribute to chronic lower limb ischemia. This study (conducted in phases I–III) aimed to identify health problems and assess patients’ quality of life with chronic lower limb ischemia treated in one Polish clinical center. Methods: The Polish version of the Vascular Life Quality Questionnaire scale (consisting of five domains (subscales)) was used to assess quality of life. Phase I was conducted in the Outpatient Clinic and included 122 patients, 38 women and 84 men, aged 41 to 88 years, with chronic lower limb ischemia. Phase II was conducted only among those (n = 88) qualified for revascularization treatment (the study was conducted preoperatively), and phase III took place six months after the revascularization procedure. Results: The primary health problem was lower limb pain and limited physical activity. With the age of the patients, the quality of life in the “symptoms” subscale deteriorated. Independent variables such as gender, marital status, level of education, and place of residence did not significantly affect the quality of life of the patients studied. Moreover, statistically significant differences in the quality of life were observed between the patients before and after revascularization treatment in the overall assessment and each area of the VascuQol scale. Conclusions: Based on our results, we stated that there is still a need to educate these patients about proper health behaviors because a large group of patients still have modifiable risk factors for the development of atherosclerosis. They require a multidisciplinary and individual approach.

## 1. Introduction

Quality of life, i.e., subjective and objective perception of one’s health and disease perception, is one factor that determines or may indicate the direction of treatment and management of patients with chronic lower limb ischemia. The symptoms of the disease often lower the patient’s quality of life and, in the advanced stage, can result in limb amputation and even patient death [1,2].

Lifestyle factors and genetic predisposition significantly influence the prevalence of lower limb ischemia [2,3,4,5]. It has been observed that despite introducing health education programs highlighting the negative impact of tobacco smoking, low physical activity, and poor eating habits leading to obesity, overweight, diabetes, and hypercholesterolemia, this group of patients often fails to adapt to medical recommendations and make necessary lifestyle changes. Hence, it underscores the need for patient education and support in making lifestyle changes, especially for the lower limb ischemia group, where the mentioned factors are significantly contributing.

Chronic lower limb ischemia manifests itself primarily with pain. Depending on the stage of the disease, the pain appears first during movement (characteristic intermittent claudication, where walking a dozen or so meters causes severe pain, and rest causes it to subside) and later also during rest. According to the studies, see [5], as many as 70% of patients with lower limb ischemia were accompanied by pain. The authors of the mentioned study emphasized the negative impact of pain on patients’ quality of life. It was found that in most respondents, pain, to some extent, led to a loss of control over part of their lives, contributed to limitations related to household chores, or prevented driving a car. In addition, long-term pain can have further consequences, such as insomnia leading to a feeling of constant fatigue, frustration, and helplessness. The reaction to pain in patients can be very diverse, often determined by previous life experiences and psychosomatic conditions. The degree of pain intensity, the ability to predict pain symptoms, and whether the patient can control them also significantly affect the perception of lower limb ischemia, which not only causes physical suffering in the patient but also affects their psyche. Pain and trophic changes lead to the isolation of patients, exclusion from social life, and make social contact difficult [2,3,4,5].

Moreover, numbness, swelling, coldness of the limbs, pale skin, loss of hair, thin, parchment skin, development of skin ulcers, and necrosis are other symptoms characteristic of this disease [2,3,4,5].

Therefore, it is essential to adapt the treatment (conservative, pharmacological, and surgical/endovascular) to the stage of the disease, the symptoms presented, and its impact on the patient’s psychophysical condition [1,2,5,6].

By determining the individual quality of life in the entire aspect of physical and psychosocial factors, doctors, nurses, physiotherapists, and psychologists can help best by adapting the treatment program to the particular patient’s needs [7].

The study’s main objective was to determine health problems and assess patients’ quality of life with chronic lower limb ischemia.

Based on the main objective of the study, the following research questions were adopted:

1. To learn about health problems and patients’ quality of life with chronic lower limb ischemia (study’s phase I—during the recruitment for the study in the Outpatient Clinic).
2. To check if the demographic variables and the progression of the patient’s disease affect the quality of life of Polish patients with chronic lower limb ischemia.
3. To answer the question of the quality of life of patients with chronic limb ischemia qualified for surgery (study’s phase II—preoperative period, before the revascularization).
4. To analyze patients’ quality of life with chronic lower limb ischemia 6 months after revascularization procedures (study’s phase III—6 months after revascularization in the Outpatient Clinic).

## 2. Material and Methods

This study was conducted at the General and Vascular Surgery Outpatient Clinic and the Department of General and Vascular Surgery Clinic in the Transfiguration of Our Lord Clinical Hospital of the Poznan University of Medical Sciences, Poland.

The physician determined the severity of chronic lower limb ischemia—peripheral arterial disease (PAD) based on the Fontaine scale and ankle-brachial index (ABI) measurement—as a primary non-invasive tool for the initial diagnosis. The ABI test was performed supine and lasted about 15 min. It involved measuring the ratio of systolic blood pressure values in the lower limbs (ankles) to systolic blood pressure values in the arms.

The study was performed in three phases. Phase I of the study was held in the Outpatient Clinic among consecutively registered patients with chronic lower limb ischemia and included 122 patients (38 women and 84 men) aged 41 to 88. The condition for qualification for the study was to provide patients’ informed consent after discussing the course of the study. When qualifying for the study, the following exclusion criteria were considered: previous lower limb amputation, lack of independence in movement, type 1 or 2 diabetes mellitus, acute lower limb ischemia, and stage IV of the chronic lower limb ischemia disease according to the Fontaine scale [6].

Phase II of the study was conducted in the preoperative period in the Outpatient Clinic among the same recruited patients, but only those qualified for revascularization treatment were selected (n = 88). Indications for revascularization were following current recommendations and included ischemia-threatening limb loss (Fontaine stage III and IIB with a short claudication distance, and when the claudication distance prevented professional work or self-care and conservative treatment proved ineffective). It should be underlined here that the indication for revascularization is also Fontaine stage IV disease, but this was an exclusion factor from this study.

Phase I and II of the study were held from 18 March 2022, to 10 June 2023. Phase III was performed 6 months after the procedure in the Outpatient Clinic.

The Polish version of the Vascular Quality of Life Questionnaire (VascuQol) scale was used to assess the quality of life. It consisted of 25 closed-ended questions presented in detail [8]. The questions covered five domains (subscales): (a) three physical aspects, such as pain (4 questions), symptoms (4 questions), and physical activities (8 questions); and (b) two psychological items, such as social (2 questions) and emotional (7 questions). The answer to each question was rated on a 7-point scale, where 1 indicated the worst and 7 was the best rating. After summarizing the items responses, an overall score and scores of five domains were generated.

Next, the patients completed the survey questionnaire. It consisted of socio-demographic characteristics, lifestyle, and comorbidities, including information on the dependent variables: age, gender, education, place of residence, marital status, and risk factors (information collected during the interview), such as smoking, hyperlipidemia, and hypertension. Cigarette smoking was considered regular smoking if the respondents indicated that they had smoked one pack of cigarettes per day for at least the last 5 years. Hyperlipidemia was assessed based on the results of laboratory tests from the previous month that the patient had at their disposal or on the fact of the use of anti-lipid drugs. In turn, hypertension was determined based on the patient’s history and/or the fact of the use of antihypertensive medications. In addition, the patients were asked, “Do the listed aspects of the VascuQoL scale constitute a serious health problem for you?”. All these issues are presented in Table 1 and Table 2, along with the results (Section 5).

## 3. Ethics

The study was conducted following the Declaration of Helsinki and approved by the Clinical Research Department of the Poznan University of Medical Sciences, registered under reference number 37/2021. Participation in the survey was voluntary and anonymous. All participants in the study gave their informed consent to participate. The informed consent form contained information about the study, its purpose, the method of answering the questions, and the possibility of withdrawing from the study at any time without suffering consequences.

## 4. Statistical Analysis

Qualitative variables and answers to the questions from the survey questionnaire and the question: Do the listed aspects of the VascuQoL scale constitute a serious health problem for you? were described in the study using the number (n) and frequency (%). On the other hand, measurable variables were described using basic parameters such as arithmetic mean, standard deviation, median, and minimum and maximum value.

The Shapiro–Wilk test was used to check the normality of distribution for measurable variables, and the condition of homogeneity of variance was used with Levene’s test.

Nonparametric tests were used for the quality of life variable and age because they did not have a normal distribution as follows:

- The Mann–Whitney U test was used to check the significance of differences in the level of quality of life in two groups;
- The Kruskal–Wallis test to check the significance of the difference in the level of quality of life in three groups;
- The Spearman rank correlation coefficient significance test was used to examine the correlation between measurable variables.

The calculations in the study were performed using the statistical package STATISTICA 10 PL. The results were considered statistically significant for *p* < 0.05.

## 5. Results

The demographic and clinical characteristics collected in the Outpatient Clinic showed that a more significant number of patients were males. The average age of men was 68.8 ± 8.9 years and women 68.7 ± 9.2 years. Most study participants lived in the city and were married. Half of the study participants had a high school education. Regular smoking of cigarettes was recorded in over 90% of the participants, and smoking in the past was found in the rest. The mean value of the ankle-brachial index was 0.80 ± 0.16. About 40% of the examined arterial occlusions at the level were diagnosed as popliteal and femoral occlusions. For detailed information, see Table 1.

The VascuQoL values range from 1 to 7, with higher values indicating better QoL.

A serious health problem for over 80% of the respondents was pain, physical activity, emotional functioning, and symptoms related to the disease.

The mean values describing the quality of life in the VascuQol scale studied five domains that indicated an average quality of life of the responders; the highest average value was reached by patients in the “Symptoms” domain, and the lowest in the “Physical activity” domain (see Table 2).

The Spearman rank correlation coefficient significance test showed a significant correlation between the age of the patients and the quality of life only for one subscale, “Symptoms” (*p* = 0.0303). This correlation was inverse with a weak strength (Rs = −0.196). The older the patients were, the lower the quality of life in the “Symptoms” subscale was.

Independent variables such as gender, marital status, level of education, and place of residence did not have a statistically significant impact on the quality of life of patients with chronic lower limb ischemia (see Table 3).

The mean values of the overall quality of life and in the individual domains of the VascuQoL scale were statistically lower (*p* < 0.0001) in the patients who were classified to Fontaine stage IIB and III compared to patients who were classified to Fontaine stage IIA (see Table 4).

Out of 122 patients, 88 were qualified for surgery. The mean values of the overall quality of life and in all domains of the VascuQoL scale were statistically higher in the study participants within 6 months of the revascularization procedure compared to patients’ quality of life in the preoperative period. Patients’ quality of life before revascularization treatment and after 6 months is presented in Table 5.

## 6. Discussion

This single-center study showed that chronic lower limb ischemia reduces quality of life. In the studied group of Polish patients, none of the five VascuQol domains used to assess quality of life achieved the maximum number of points, with the lowest values in the pain and physical activity domains. Conversely, the highest mean values were obtained in symptoms, social functioning, and emotional domains, which indicated that patients feel a minor impact of the disease on their mental state and social sphere.

The overall mean quality of life score according to the VascuQol scale was 3.29 ± 1.27, which is similar to the results obtained by Nordanstig et al. [9] and Frans et al. [10], where the total quality of life score was 3.04 ± 0.97 [10]. The results in the individual domains were also distributed similarly, with the lowest scores for “Physical Activity” and “Pain” and the highest for “Emotions”, “Symptoms”, and “Social Functioning”. The importance of our research is evident in the use of the VascuQoL scale, which was also used to indicate low quality of life by Jens et al. [11], Landry et al. [12], and Powell et al. [13]. Rha et al. [14] took this approach in their multicenter study among Korean patients with peripheral arterial disease (PDA). They used the EuroQoL-5 dimensions-3-level (EQ-5D-3L) survey-Korean version to assess the quality of life, conducting it twice, at the beginning and after 6 months. Their results indicated a not-so-worst assessment of the quality of life (scores 67.49 ± 18.29 and 71.56 ± 16.33, respectively (with the best score of 100 and the worst score of 0)). Siracuse et al. [15] analyzed the quality of life in patients undergoing lower limb revascularization using three questionnaires: VascuQoL; a 12-item short-form survey (SF-12), including the utility index score (SF6D-R2), mental composite scale (MCS), and physical composite scale (PCS); and EQ-5D about four grades of the WIfI (wound, ischemia, foot infection). In all scales, patients presented a low quality of life, especially in their mental state, compared to their physical state. Corriere et al. [16] indicated a lower quality of life using the shortened version of VascuQoL-6, especially in patients after numerous therapeutic interventions. Nordanstig et al. [17] used this version to evaluate PAD revascularization procedures. The studies by Paplaczyk et al. [18] revealed that patients with chronic lower limb ischemia assessed their overall quality of life (WHOQOL-BREF—World Health Organization Quality of Life-BREF) as average or poor. Belowski et al. [8] also obtained similar quality of life values in patients suffering from chronic lower limb ischemia, both in the VascuQol scale and the SF-36 and EQ-5D-3L. In addition, Peters et al. [19], the older adults assessed their quality of life in the mental, physical, and social areas as much worse before medical interventions.

Our study showed that gender, marital status, education, and place of residence do not affect the quality of life. Similar results were obtained by Paplaczyk et al. [18] with schooling and place of residence. However, different results were obtained concerning marital status—patients in stable relationships demonstrate a significantly higher quality of life than single people. Women also had a lower quality of life than men. Powell et al. [13] obtained identical data on gender, while Corriere et al. [16] found the opposite—men had a worse quality of life than women. In Peters et al. [19] and Perlander et al. [20], gender did not affect quality of life, although the studies were conducted on people over 70. Our research showed that the older the patient is, the lower their quality of life is, especially in the symptoms domain. Paplaczyk et al. [18] indicated that the age of patients did not affect their quality of life. Still, when age was associated with fitness, it turned out that patients with lower fitness had a significantly lower quality of life. According to Powell et al. [13], younger age predisposed to a lower quality of life in the mental area. The analysis of our study results showed that patients with chronic lower limb ischemia are burdened with other diseases that can significantly affect their functioning. The comorbidities indicated by the patients included arterial hypertension and hypercholesterolemia. Frans et al. [10] also noted the above comorbidities in the group of study participants, as well as coronary artery disease, chronic obstructive pulmonary disease (COPD), a history of transient ischemic attack (TIA), stroke, and renal failure. Hageman et al. [21] analyzed the co-occurrence of diseases such as obesity, myocardial infarction, congestive heart failure, TIA, and COPD.

However, no studies were found on the influence of co-occurring diseases on the quality of life in patients with PDA. Only the studies by Corriere et al. [16] discussed this aspect, although they did not show a relationship between co-occurring diseases and quality of life.

Cigarette smoking is known to stimulate clot formation via platelet activation, increased tissue factor, increased blood viscosity caused by catecholamines, thromboxane A2-induced coronary artery blockage, and impaired prostacyclin production. In addition, it contributes to endothelial damage and atherosclerosis development [22,23]. There is, therefore, no doubt that this factor plays a key role in both the onset and severity of lower limb ischemia. However, in our study, we did not analyze the influence of cigarette smoking on the quality of life. Almost all studied patients (n = 112) were current smokers, and ten were previous smokers, so we had no groups to compare. However, such an analysis was undertaken by Powell et al. [13] and Siracuse et al. [15]. They indicated, based on their studies’ results, that smoking reduces the quality of life.

Moreover, our study did not assess the influence of the body mass index (BMI) (26.95 ± 4.33) on the quality of life. Such an analysis was conducted by Łagoda et al. [5], who did not note a significant difference in the level of BMI by age, education, and place of residence of the subjects [5].

The main symptom of chronic lower limb ischemia is intermittent claudication, which is accompanied by pain during walking. The analysis of our study showed a significant difference in the quality of life of patients covering a distance of up to 200 m (IIB—stage, according to Fontaine) and patients covering a distance of over 200 m without pain (IIA—stage, according to Fontaine). The overall quality of life and quality of life for each of the five domains of the VascuQol scale were higher in people covering a distance longer than 200 m without pain. Paplaczyk et al. [18] also proved that the circumstances of pain occurrence, according to the Fontaine scale, affect the quality of life of patients with chronic lower limb ischemia [18,21]. Also, Paplaczyk et al. [18] proved in their studies that when the intensity of the pain experienced was higher, it correlated with a lower quality of life in the physical activity domain. Rha et al. [14] analyzed the medical records of Korean patients with PDA. They found that 11.4% of the study population had used pain clinic services, and 5.8% had been hospitalized in pain management units. In the studies of Siracuse et al. [15] and Powell et al. [13], the use of opioid drugs was associated with worse results of the quality of life on the VascuQol scale.

In our study, statistically significant differences in the quality of life were observed in the overall assessment and each of the studied domains of the VascuQol scale between patients before and after revascularization treatment. Similar results were also found in the studies of Belowski et al. [8] and Corriere et al. [16]. Corriere et al. indicated that revascularization procedures can significantly improve the quality of life. Surgical procedures permanently enhance the quality of life (especially in the physical and mental domains) of older people (>70 years of age). In contrast, endovascular procedures were only crucial for the changes in the physical domain. In our study, we did not perform analyses regarding differences in revascularization procedures (surgical or endovascular).

Steunenberg et al. [24], Frans et al. [10], Perlander et al. [20], and Nordansiga et al. [9,17] also demonstrated a positive effect of vascular procedures on the quality of life.

Moreover, Hageman et al. [21] analyzed the effect of supervised exercise therapy (SET) on the quality of life and achieved its improvement 3 months after the beginning of the treatment. Rha et al. [14] assessed the effect of pharmacotherapy and showed a significant impact on patients’ increased quality of life. In Peters et al. [19], conservative treatment did not change the patient’s assessment of their quality of life, in contrast to the results of Steunenberg et al. [24]; their studies showed that patients achieved a significant improvement in their quality of life in the physical area.

Chronic limb ischemia has an adverse impact on quality of life. Assessment of PAD treatment outcomes based on objective clinical parameters does not always allow for a reliable assessment of pain, discomfort, and social and emotional aspects. Symptoms of the disease often reduce the patient’s quality of life and, in advanced stages, may result in limb amputation or even death of the patient. Hence, studies such as ours can help personalize treatment and better understand the needs of this group of patients. This is important because epidemiological data indicate that approximately 1.5% of people over 50 years of age have been diagnosed with atherosclerosis of the lower limb arteries; in 3%, the disease is not diagnosed despite the occurrence of characteristic symptoms, while 11% are asymptomatic patients. In Poland, it is estimated that approx. 30 thousand people report to a doctor every year due to atherosclerotic ischemia of the lower limbs. This number is increasing year by year [25].

As the number of people with chronic diseases increases, it is imperative to ensure that they achieve the best quality of life. To reach this, first, an assessment of the disease’s impact and medical interventions’ effects should be made using various quality-of-life scales [26].

There are specific scales for many disorders. They assess a narrower range than non-specific instruments and evaluate patients’ quality of life with a given disease. These scales tend to be more sensitive to change; for example, there exists in rheumatoid arthritis [27,28], lower limb ischemia [8], or cardiovascular disease [29]. The strength of our study was evaluating the quality of life in lower limb ischemia patients using a validated scale focusing on key areas of functioning, physical, mental, and social, as well as subjective patient assessment, to assess the quality of life in lower limb ischemia patients.

The main potential limitation of our study was related to the small study population. The study omitted information on employment and income, which may be considered a limitation. Moreover, some factors were not taken into account, such as whether the patients changed their lifestyle after the surgery, nor was the psychological condition of the patients analyzed in the pre-operative period (e.g., whether they suffer from depression or anxiety disorders). It should be noted that people affected by chronic lower limb ischemia are physically and mentally burdened. Hence, the disease could cause anxiety, depression, and social isolation in patients as a consequence of the rapidly appearing pain and, consequently, the inability to leave the house—for shopping, work, and meetings with friends. Depression could limit the mobilization to walk and treatment, and this, in turn, affects worse physical functioning and cognitive aspects [30], and this was not taken into account in our study. Other limitations of our study included some clinical aspects. First, we did not examine the ABI after surgery; the physician determined the ABI only once on admission to the hospital. In addition, we haven’t focused on the way of revascularization treatment and its impact on quality of life.

Many of the studied patients with lower limb ischemia were still burdened with causative factors like tobacco smoking, hypertension, and hyperlipidemia. Their disease had a negative impact on their quality of life, but gender and sociodemographic factors did not affect it. The overall assessment of quality of life was better after revascularization treatment.

Based on our results, we stated that there is still a need to educate these patients about proper health behaviors because a large group of patients still have modifiable risk factors for the development of atherosclerosis. They require a multidisciplinary and individual approach.

## Figures and Tables

**Table 1:** Demographics and clinical characteristics and risk factors in the patient population collected in the Outpatient Clinic.

Variables	Mean ± SD or No. (%) (n = 122)
Demographics:	
Age, years	68.8 ± 8.9
Sex:	
Male	84 (68.9)
Female	38 (31.1)
Education:	
Elementary school education	17 (13.9)
Professional education	38 (31.1)
High school education	61 (50.0)
Higher education	6 (4.9)
Place of residence:	
City	81(66.4)
Country	41(33.6)
Marital status:	
Single	10 (8.2)
Married	81(66.4)
Divorced	5 (4.1)
Widowed	26 (21.3)
Risk factors:	
Regular smoking ^#^	112 (91.8)
Smoking in the past	10 (12.2)
Non-smokers	0
Hyperlipidemia (in an interview) *	75 (61.5)
Hypertension (in an interview) **	97 (79.5)
Ankle-brachial index (ABI) ^	0.80 ± 0.16
Classified according to Fontaine:	
IIA	36 (29.5)
IIB	36 (29.5)
III	50 (41.0)
Degree of arterial obstruction:	
Leriche syndrome	32 (26.2)
Popliteal, femoral occlusion	48 (39.3)
Distal obstruction	42 (34.5)

^#^ Regular smoking—the respondents indicated that they had smoked one pack of cigarettes per day for at least the last 5 years; * hyperlipidemia—based on the results of laboratory tests from the previous month that the patient had at their disposal or on the fact of the use of anti-lipid drugs; ** hypertension—determined on the results of measurements available from the patient’s history and/or the fact of the use of antihypertensive medications; ^ ankle-brachial index (ABI) indicated as reduced when <0.9.

**Table 2:** Health problems and quality of life according to the Vascular Quality of Life Questionnaire in patients with lower limb ischemia (n = 122)—(Phase I).

VascuQol Scale Domains	Mean ± SD	Median	Do These Indicated Aspects Constitute a Serious Health Problem for You? Answer YES n (%)
1. Physical activity (exercise or playing sports; walking distance has increased; ability to walk; ability to climb stairs; ability to do routine household work; range of activities; ability to go shopping or carry bags; walking distance became less)	3.14 ± 1.38	3.13	112 (91.8)
2. Pain (pain during walking, pain at night, pain at rest, the amount of discomfort)	3.22 ± 1.25	3.00	122 (100)
3. Symptoms (cold feet; tired or weak legs; pins, needles, or numbness; ulcers and sore)	3.63 ± 1.42	3.50	87 (71.3)
4. Emotional (worry about injuries; concerns about being housebound; concerns about having poor circulation in the legs; frustration about problems caused by poor circulation in the legs; feelings of guilt about relying on friends or relatives; worries about being in danger of losing a part of leg or foot; being depressed about poor circulation in the legs)	3.27 ± 1.28	3.07	106 (86.9)
5. Social (restricted in spending time with friends or relatives; ability to participate in social activities)	3.43 ± 1.60	3.50	48 (39.3)
The overall quality of life	3.29 ± 1.27	3.24	

**Table 3:** Correlation of the age of the respondents with individual domains of quality of life (phase I).

Pair of Variables	n	Rs	*p*
Age (years) and Physical Activity (points)	122	−0.091	0.3178
Age (years) and Pain (points)	122	−0.087	0.3411
Age (years) and Symptoms (points)	122	−0.196	0.0303
Age (years) and Emotions (points)	122	−0.162	0.0750
Age (years) and Social Functions (points)	122	−0.173	0.0565
Age (years) and Overall Assessment (points)	122	−0.152	0.0951

Rs—Spearman’s rank correlation coefficient value; *p* < 0.05 is considered statistically significant.

**Table 4:** Quality of life of the patients classified according to the Fontaine stage (phase I).

VascuQol	Classified According to Fontaine	n	Mean	SD	Median	Minimum	Maximum	Z	*p*
Physical Activity	IIA	36	4.10	1.26	4.13	1.75	6.25	−6.33	<0.0001
	IIB, III	86	2.43	0.98	2.31	1.00	4.50		
Pain	IIA	36	3.95	1.18	4.00	2.00	7.00	−5.52	<0.0001
	IIB, III	86	2.67	1.00	2.50	1.00	5.00		
Symptoms	IIA	36	4.27	1.21	4.75	2.00	6.50	−4.28	<0.0001
	IIB, III	86	3.15	1.39	3.00	1.00	6.00		
Emotions	IIA	36	3.96	1.12	3.93	2.29	7.00	−5.31	<0.0001
	IIB, III	86	2.76	1.15	2.43	1.00	5.57		
Social Functions	IIA	36	4.30	1.62	4.00	1.50	7.00	−4.86	<0.0001
	IIB, III	86	2.79	1.24	2.50	1.00	6.00		
Overall Assessment	IIA	36	4.08	1.14	4.22	2.24	6.68	−5.89	<0.0001
	IIB, III	86	2.70	1.02	2.56	1.04	4.76		

The Mann–Whitney U test was used for the analysis; the VascuQoL ranges from 1 to 7, with higher values indicating better HRQoL; *p* < 0.05 is considered statistically significant.

**Table 5:** Quality of life patients before revascularization treatment and after 6 months.

VascuQol	Before/After	Mean	SD	Median	Minimum	Maximum	Z	*p*
Activities	Before	2.88	1.32	2.69	1.00	6.25	−3.08	0.0021
	After	3.69	1.34	3.56	1.13	6.00		
Pain	Before	3.02	1.24	2.75	1.00	7.00	−2.51	0.0121
	After	3.61	1.17	3.63	1.50	6.00		
Symptoms	Before	3.45	1.46	3.25	1.00	6.50	−1.97	0.0491
	After	3.99	1.28	4.38	1.00	6.00		
Emotional	Before	3.09	1.34	2.86	1.00	7.00	−2.66	0.0077
	After	3.64	1.08	3.86	1.43	5.57		
Social	Before	3.14	1.52	3.00	1.00	7.00	−3.08	0.0020
	After	4.04	1.59	4.00	1.00	7.00		
The overall quality of life	Before	3.07	1.27	2.88	1.04	6.68	−2.83	0.0046
	After	3.74	1.15	3.86	1.84	5.36		

The Mann-Whitney U test was used for the analysis; the VascuQoL ranges from 1 to 7, with higher values indicating better HRQoL; *p* < 0.05 is considered statistically significant.

## Data Availability

The data are not publicly available due to data privacy regulations. The data presented in this study are available upon request from the corresponding author.
